# Review of Optical Imaging in Coronary Artery Disease Diagnosis

**DOI:** 10.3390/jcdd12080288

**Published:** 2025-07-29

**Authors:** Naeif Almagal, Niall Leahy, Foziyah Alqahtani, Sara Alsubai, Hesham Elzomor, Paolo Alberto Del Sole, Ruth Sharif, Faisal Sharif

**Affiliations:** 1 College of Medicine, University of Galway, H91 TK33 Galway, Irelandfaisal.sharif@universityofgalway.ie (F.S.); 2 Cardiology Department, Neom Hospital, Sharma 49626, Saudi Arabia; 3 Cardiac Technology Department, Imam Abdulrahman Bin Faisal University, Dammam 31441, Saudi Arabia

**Keywords:** optical coherence tomography (OCT), coronary artery disease (CAD), fractional flow reserve (FFR), intravascular ultrasound (IVUS), plaque morphology, coronary imaging, OCT-based FFR

## Abstract

Optical Coherence Tomography (OCT) is a further light-based intravascular imaging modality and provides a high-resolution, cross-sectional view of coronary arteries. It has a useful anatomic and increasingly physiological evaluation in light of coronary artery disease (CAD). This review provides a critical examination of the increased application of the OCT in assessing coronary artery physiology, beyond its initial mainstay application in anatomical imaging. OCT provides precise information on plaque morphology, which can help identify vulnerable plaques, and is most important in informing percutaneous coronary interventions (PCIs), including implanting a stent and optimizing it. The combination of OCT and functional measurements, such as optical flow ratio and OCT-based fractional flow reserve (OCT-FFR), permits a more complete assessment of coronary stenoses, which may provide increased diagnostic accuracy and better revascularization decision-making. The recent developments in OCT technology have also enhanced the accuracy in the measurement of coronary functions. The innovations may support the optimal treatment of patients as they provide more personalized and individualized treatment options; however, it is critical to recognize the limitations of OCT and distinguish between the hypothetical advantages and empirical outcomes. This review evaluates the existing uses, technological solutions, and future trends in OCT-based physiological imaging and evaluation, and explains how such an advancement will be beneficial in the treatment of CAD and gives a fair representation concerning other imaging applications.

## 1. Introduction

Optical Coherence Tomography [OCT] is an intravascular imaging modality based on light that can produce cross-sectional images of the coronary arteries in high resolution [1]. These near-IR images allow for fine anatomical characterization of complex intravascular plaque and complete coronal stent evaluation [2]. OCT has been integrated as a valuable resource in interventional cardiology, where its high resolution in the axial dimension is valuable in precise coronary anatomical imaging. Although commonly associated with its high-resolution abilities in describing anatomical details, its clinical value can be better comprehended by merging the understandable benefits and pitfalls of using it over the others [1,3]. In addition to assessing coronary anatomy, OCT is gaining popularity as an instrument for examining coronary physiology, derived metrics such as OCT-based FFR, and optical flow ratio [4,5]. Physiological diagnostic assessment is paramount to determining the importance of a coronary stenosis that, when added to anatomic information, makes decisions on revascularization procedures, including coronary artery bypass grafting [CABG] and percutaneous stent implantation [6]. The imaging of the coronary has evolved and gained momentum with the introduction of other imaging modalities, having enhanced length and resolution. OCT is a type of imaging first used in intravascular medical imaging [2]. It has further developed, and its use in coronary physiology is growing, specifically in guiding the ideal plaque morphology, assessing stent apposition, and helping in conducting complex interventions [7,8].

This review aims to provide an in-depth review of the role of OCT in the physiological assessment of the coronary arteries. It will also critically examine the science of OCT imaging, its clinical uses in coronary physiology, comparative evaluation with other modalities in terms of fairness, its own limitations, and prospects towards novel directions at the clinical level as well as on the research front. Last, this review will describe the general role of OCT-based physiological assessment in the management of coronary artery disease in a comprehensive manner, substantiating the claims with high-level evidence when present.

## 2. OCT in Coronary Atherosclerosis Assessment

The high-resolution cross-sections that OCT can reveal provide access at a microscopic level, viewing plaque morphology and providing detailed information of coronary atherosclerosis compared to that of angiography [2]. It provides this amount of detail that makes physicians notice the presence of complex features that indicate plaque vulnerability, including thin-cap fibroatheromas [TCFA], lipid-rich necrotic cores, and fibrous caps [2]. Moreover, the use of OCT in the assessment of plaque morphology can give a better insight into underlying destructive pathology, which facilitates the differentiation between stable and unstable plaques [4]. The presence of a specific component of the plaque can be detected by OCT and thus increase diagnostic accuracy, helping to identify the risk level and plan the treatment [9].

The OCT employs the near-infrared spectrum and catheters to provide high resolution of the inside of hard-to-see coronary arteries, making it ten times more spatially clear than intravascular ultrasound [IVUS] [1,3]. At this high resolution, OCT can examine microstructures of plaques in arteries, allowing characteristics to be distinguished, including fibrous tissue, lipid-filled components, and the calcified deposits [10,11]. Signal-enriched areas usually consist of homogeneous fibrous plaques. Fibrocalcific ones appear as foci of low signal intensity with well-defined borders and lipid-rich areas as an area of low or no signal with indistinct borders [10,11]. TCFA is fibrous caps less than 65 microns in thickness covering large lipid cores, which OCT can detect. Such plaques are believed to be high-risk plaques liable to rupture, which can initiate acute coronary syndromes [ACS] [9]. The method also makes it possible to detect a rupture in the plaque, thickening of the wall of the arteries, and the appearance of thrombi, which allows for the identification of the causes of coronary events [1]. The high-resolution images made by OCT allow for the quantification of fibrous cap thickness and define the microstructure of plaques, which points to its superiority in risk prediction over IVUS under some conditions [1,3].

OCT was useful in classifying stable and unstable plaques, and it has indicated dynamic processes that relate to wound healing and destabilization that may promote atherosclerotic disease [4]. It helps in finding significant information, such as the existence of macrophages and neovascularization, which is of importance to plaque vulnerability and inflammation [12]. Although OCT can observe tissue up to 1–3 mm in depth, it can still be applied in research and clinical practice because it provides a detailed overview of the top superficial layers of plaques [13]. Thus, the capability of OCT to visualize the coronary of the internal surface of the arteries and determine the type of plaques with accuracy has been game-changing in the methodology to investigate coronary atherosclerosis and efficient ways to manage patients with CAD [7,14].

### Limitations of OCT in Atherosclerosis Assessment

Despite its high resolution, OCT has several inherent limitations that must be considered:

Limited Penetration Depth: OCT’s shallow tissue penetration [1–3 mm] means it cannot visualize the full extent of plaque burden, particularly in large, eccentric plaques or deeply embedded lesions. This contrasts with IVUS, which offers greater penetration [11].

Requirement for Blood Clearance: Optimal OCT image acquisition necessitates complete blood clearance from the vessel lumen, typically achieved by flushing with saline or contrast. This can be challenging in tortuous vessels, in patients with poor renal function [due to contrast use], or in those with significant collateral flow [15].

Inability to Image Through Dense Calcification: Dense calcified plaques cause significant signal attenuation and shadowing, obscuring the underlying vessel wall and limiting comprehensive assessment of plaque morphology and stent apposition in heavily calcified lesions [11].

Cost and Operator Dependence: OCT systems and single-use catheters can be more expensive than IVUS, and the interpretation of OCT images requires specialized training and experience [3].

**Figure 1:**
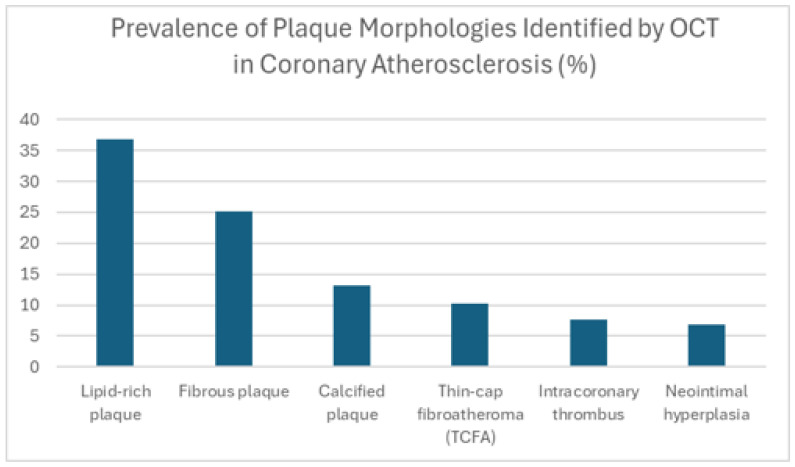
Prevalence of plaque morphologies identified by OCT in coronary atherosclerosis [%].

This graph [Figure 1] provides exemplary data on the prevalence of various forms of plaque in the coronary artery, illustrating the heterogeneity of the disease. In this illustrative dataset, lipid-rich plaques are shown to be encountered most regularly. From a clinical perspective, a lipid-rich plaque with a thin cap [TCFA] is considered vulnerable and more likely to rupture, which can lead to acute coronary events. Stable fibrous plaques are the second most common type at 25%, but, depending on their size, they can also contribute to arterial narrowing. Calcified plaques, occurring in 15% of cases, can complicate PCI procedures due to their rigidity. The presence of thin cap fibroatheromas [TCFAs] in 10% of cases is significant, as these characteristics warrant close attention. The smaller numbers of intracoronary thrombus [8%] and neointimal hyperplasia [7%] reflect the dynamic nature of thrombus formation [acute events] and hyperplasia [long-term response post-stenting]. Overall, these illustrative findings highlight that OCT adds value by describing the multifaceted pathology of coronary artery disease, offering a more detailed assessment compared to angiography’s primary focus on luminal dimensions.

OCT is also used to determine vulnerable plaques, which have a substantial effect on the determination of risk and choice of treatment in coronary artery disease. OCT can be critical in selecting persons at higher risk of acute coronary events since it permits the likelihood of rupture to be recognized when plaques are more likely to break [9]. It is based on this knowledge that the targeted preventative actions can be pursued, including intensive risk factor management and pharmacological treatment directed at making vulnerable plaques stable. Finally, OCT-guided therapies have the potential to deliver personalized regimens, including optimum stent placement and optimization that could potentially improve clinical outcomes during extended tenure in coronary artery disease patients [7].

## 3. Physiological Assessment of Coronary Lesions

Anatomical and functional information is now included in the physiological assessment of coronary lesions. Such an approach offers a more comprehensive tool for assessing lesion severity “see Table 1 below”. This includes the incorporation of fractional flow reserve [FFR], a method of determining the hemodynamic relevance of coronary stenoses, into OCT [6]. Although OCT can be used to identify the characteristics of vulnerable plaque, like thick lipid pools or thin-cap fibroatheromas [2], FFR can be used to evaluate the lesion-related physiological effect and prioritize types of revascularization strategies based on the quantification of ischemia burden [6]. A combination of such methods enables clinicians to outline the severity of lesions more accurately and identify hemodynamically significant stenoses. The hybrid approach enables a more accurate diagnosis of the character of the lesions and their impact on the blood circulation in the coronary arteries, which can lead to more targeted forms of therapy.

This requires that the physiology of the lesions be observed so that one understands the impact on blood flow and whether there is a need for revascularization. On the one hand, some researchers believe that FFR, which measures a parameter during angiography with the help of a pressure wire, can become a stable tool for preparing ischemia developing due to a coronary lesion [6]. Nevertheless, FFR using wire requires insertion of other catheters and vasodilator medication [e.g., adenosine], extending procedure time and causing pain to the patient [9].

Currently, OCT-based structural features such as minimal lumen area [MLA], plaque burden, and lesion length are applied to characterize coronary constriction. These parameters of OCT can give good anatomical information but are not direct measures of ischemic significance. Incorporated into the assessment of ischemia and FFR, these results of OCT anatomical parameters may supplement clinical decisions as an alternative approach to anatomical evaluation [1,9].

The most recent development is the appearance of OCT-based fractional flow reserve [OCT-FFR], where instead of the pressure wires and a known vasodilator, the pressure gradients are computed via computerized modeling of the OCT lumen imaging and used to find ischemia-related lesions [5]. In several clinical trials, OCT-FFR has been closely correlated with the wire-based FFR and has potential in outcome prediction after PCI [16]. Integrating the derived physiological data through OCT and anatomical details of this imaging modality, OCT-FFR is expected to increase the identification of lesions in need of revascularization [13].

Furthermore, through the assessment of OCT images, clinicians can detect so-called silent lesions bearing the signs of vulnerable plaque [e.g., thin cap fibroatheroma] that might not be hemodynamically relevant but have prognostic implications and provide risk stratification [4]. Although OCT-FFR has high potential, clinical use at present depends on uniform algorithms and expanded clinical verification across different patients. Researchers are already trying to implement OCT-FFR more widely and combine it with typical clinical practice to help patients with coronary artery disease [11].

The table above gives a demonstration of the description of significant parameters in the functional assessment of coronary lesions. OCT is effectively utilized in anatomical details, but stenosis-related ischemia is mostly evaluated by invasive pressure wire and flow wire evaluations. Fractional flow reserve [FFR] has become a solid component of revascularization interventions because of its established prognostic predictive capacity. The use of the instantaneous wave-free ratio [iFR] addresses this aspect as an additional hyperemia-free approach, which could make the assessment easier. The importance of coronary microvasculature, as described in both Coronary Flow Reserve [CFR] and Index of Microcirculatory Resistance [IMR], is of particular interest when a patient with non-obstructive CAD continues to experience angina. With properly combined anatomical data of OCT and physiological findings, it enables clinicians to have a better picture of the disease status of coronary arteries to further direct proper interventions.

## 4. Role of OCT in Coronary Imaging

OCT has a significant role in imaging of coronaries since it offers extraordinarily high-quality and detailed information concerning coronary pathophysiology. It allows for distinguishing the specifics of coronaries, including the walls of arteries, the diameter of the lumen, and atherosclerotic plaques [2]. OCT gives clinicians real-time access to high-resolution images of the endothelium that can be used to evaluate the structure and/or functioning, hence an excellent supplement to understand vascular health and possible endothelial dysfunction [15]. This feature improves the evaluation of the development of coronary artery disease and the impact of atherosclerotic plaques on the functioning of the coronary arteries.

OCT has made a significant advancement in cardiac imaging technology, whereby the microstructure of coronary arteries can be viewed in detail. The role is paramount in the context of diagnosis and ascertains treatment, as well as the mainstay of percutaneous coronary interventions [PCI], [7]. OCT application allows clinicians to detect procedural complications, including stent malposition, edge dissections, tissue prolapse, or the occurrence of thrombus, which are not always identified using conventional angiography only [14]. With clear images being delivered, OCT can guide more accurate stent deployment that has been linked to a notable decrease in adverse outcomes like restenosis and stent thrombosis [8].

The results of clinical studies, such as randomized controlled trials and meta-analyses, proved that OCT-guided PCI results in better outcomes of the procedure than angiography-guided PCI. Particularly, OCT-based guidance has been linked to increased minimal stent surface area, decreased incidence of stent thrombosis, and target lesion revascularization [TLR]. The ULTIMATE trial [NCT02172175] and the ILUMIEN III trial [NCT02621004] have exemplified these benefits in evidence with how the accurate visualization of stent expansion and apposition that OCT can provide leads to improved clinical outcomes [2,7]. OCT allows for examination of the structure of plaques and guides treatment options depending on the specificities of the plaque [17]. OCT performed post-PCI needs to be used to confirm the location, expansion, and coverage of the stent implanted, which are important to maintain vessel patency in the long run and avert adverse events [8].

Moreover, OCT can give immediate feedback to operators during PCI, potentially resulting in an improved decision process and improved outcomes of the procedure [8]. Following up on plaque healing and neointimal coverage with the modality provides information that supports ongoing monitoring and risk stratification in the long term [17]. Overall, OCT is now an integral part of interventional cardiology, contributing to more successful procedures and potentially leading to better patient outcomes in coronary artery disease [14].

**Figure 2:**
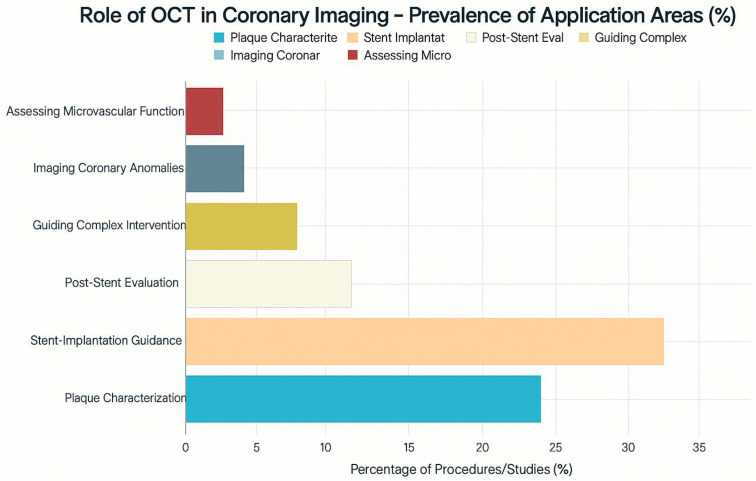
Prevalance of application areas of OCT in coronary imaging with percentages.

This graph [Figure 2] gives some of the possible applications of Optical Coherence Tomography [OCT] in coronary imaging with hypothetical data as to how often it could be applied in different circumstances. This is just to give an illustrative figure. OCT’s most common illustrative use [40% of the time] is for stent implantation guidance, providing clinicians with high-resolution details on stent placement and potential issues like incomplete stent deployment or edge dissections, which carry risks of stent thrombosis and restenosis. Plaque identification and characterization [30%] are also important uses, underscoring OCT’s ability to examine in vivo tissue and identify features of vulnerable plaque that may not be apparent on angiography. OCT can be used after stent placement [15%] to detect and assess in-stent restenosis and other long-term complications. It is also valuable in complex procedures [8%] or for imaging unusual coronary anomalies [4%]. The smaller illustrative use of OCT to measure microvascular function [3%] reflects that technology primarily provides anatomical details. This chart demonstrates that OCT contributes to interventional cardiology in multiple ways, offering valuable information about vessel wall conditions and procedural optimization.

OCT allows plaque components to be observed and the presence of lipids, calcification, and fibrous tissue [2]. This lengthy reflection on the composition of plaque can provide us with information about coronary disease that enables us to better evaluate risks and interventions. Furthermore, the technologies in OCT provide essential information on the micro vessel functionality, endothelial functions, and crowned flow reserve, all of which are significant in the advancement of insight into coronary physiology [15]. These findings not only broaden the scope of knowledge regarding coronary physiology but also affect clinical choices aimed at managing better patient outcomes in the coronary artery disease management domain.

## 5. Morphometric Assessment for Functional Evaluation

Although the morphometric functional assessment can provide anatomical information that would be invaluable, these results must be correlated with physiological measurements to effectively know the functional significance of coronary stenoses. To illustrate, the optical flow ratio is a comparatively novel metric aiming to fill some of the gaps between structural anatomy [OCT] and functional deficiency [testing, e.g., iFR and FFR] [1,4]. This technique has great advantages over the other more traditional means of assessment since it considers both the anatomical and physiological traits, thus perhaps improving the values of the diagnosis and treatment decision-making areas in the context of coronary artery disease care.

The data obtained through OCT measurement of the lumen, plaque located in the arteries, and the thickness of the fibrous cap are valuable anatomical data in terms of cardiac evaluation of the blood vessels [2]. Although parameters such as minimal lumen area [MLA] may be suggestive, it is especially important to point out that morphometry cannot conclusively prove the ischemic significance of all lesions. Functional specificity may differ across lesions, with the connection between anatomical narrowing and functional impairment being complicated and dependent on different variables [12]. Computational modeling [e.g., CFD-derived FFR] is the only method that provides functional information of anatomical data [18].

For instance, while MLA thresholds measured by OCT can accurately guide revascularization decisions for certain patient populations, such as diabetics, these are often based on correlations rather than direct functional measurements [4]. In addition to lumen measurements, OCT identifies the thickness of the plaque cap and the size of its central lipid area, which play a key role in determining the likelihood of a plaque rupture [12]. Morphometric measurements can help discover high-risk plaques, whether they are significant in terms of function or not [4]. Also, monitoring plaque development or regression over time using OCT has proven to be useful for tracking the impact of medical therapies [12].

The analysis of OCT-derived morphometric data also aids in building computational models that relate morphological measurements to hemodynamic signals to predict outcomes for patients [19]. Linking morphometric details and functional assessment increases the effectiveness of CAD management by guiding clinicians in deciding the best methods of risk assessment and treatment [4].

**Figure 3:**
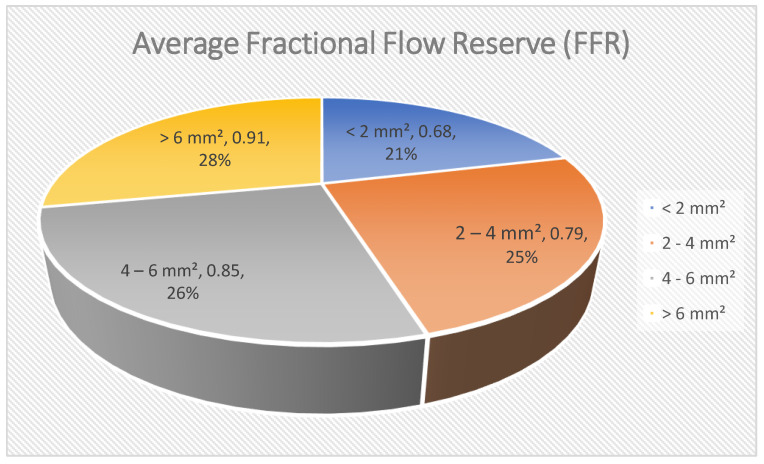
Average fractional flow reserve [FFR].

In this graph [Figure 3], illustrative data are given to show that a smaller diameter of the minimal lumen area [MLA] of the sector at OCT translates to a smaller functionally relevant obstruction as measured by FFR. The number is conceptual and not empirical. As the MLA increases, the FFR often rises and approaches the normal value of 1.0. Conversely, tight narrowing seen on angiography usually correlates with low FFR values, suggesting the lesion causes hemodynamic problems. When the MLA is the smallest [e.g., <2 mm^2^], the drop in FFR is typically the largest, which suggests that tight narrowing of the vessel can lead to ischemic issues. As MLA increases, the average FFR also increases, indicating less obstruction of blood flow through the vessel. This descriptive information indicates that MLA acquired by OCT is valuable in getting an idea of how a coronary lesion can anatomically impact the functioning of the heart. Although this relationship is not absolute and MLA does not completely indicate FFR, it can be concluded that, in many cases, anatomical characteristics can affect hemodynamic relevance. When combined with conventional angiographic and invasive functional examinations, OCT-derived anatomical measurements can be used to determine the severity of lesions, enabling them to be treated with revascularization techniques [20].

The morphometric and functional data combination allows for assessing the coronary lesions in a more specific manner so that revascularization strategies can be more effective. This combined technique allows cardiologists to target therapy with lesions with the greatest effect on coronary blood flow by distinguishing lesions that are both functionally significant and anatomically important [21].

## 6. Integration of Angiography and Intracoronary Imaging

Incorporation of angiography with intracoronary imaging devices, including OCT and IVUS, can assist in supplying augmenter information to determine an extensive assessment of the coronary lesions. Angiography offers only macroscopic appearances of the luminal narrowing and coronary anatomy, whereas OCT and IVUS offer superior diagnostic sensitivity by defining certain attributes related to the plaque composition and structure, as well as vascular remodeling [11,22]. Clinicians are thus in a better position to interpret more clearly the lesion characteristics and functional significance. The combination of the modalities, in turn, will already avoid the necessity of using each of the modalities separately, and will instead enable a more comprehensive evaluation of the coronary artery disease that will subsequently enable optimization of treatment regimens [20].

The dual approach of conducting OCT or IVUS on patients during coronary angiography gives in-depth detail about coronary artery disease by virtue of their strengths. However, angiography enables us only to visualize the exterior of a vessel, giving little insight as to the plaques inside the vessel wall, the inner tissues of the vessel, or the specific location of stent placement [1].

### 6.1. Comparison of OCT and IVUS

The balanced comparison between OCT and IVUS is essential to comprehend their roles in intracoronary imaging:

Resolution OCT provides much greater axial resolution [10–20 μm] than IVUS [100–150 μm] [1,3]. This high resolution enables OCT to resolve intricate microstructures, exactly quantify fibrous cap thickness, and measure stent strut apposition with increased accuracy.

Depth of Penetration: IVUS possesses stronger tissue penetration [5–8 mm], whereas OCT only has penetration [1–3 mm] [11]. This benefit enables IVUS to assess more effectively overall plaque burden, image the external elastic lamina [EEL], and measure vessel remodeling, especially large or severely diseased vessels that IVUS may have a lower signal throughput [23].

Blood Clearance: OCT requires complete blood clearance for optimal image acquisition, typically achieved with a saline or contrast flush. IVUS, being ultrasound-based, does not require blood clearance, making it potentially easier to use in certain clinical scenarios [15].

Imaging Through Calcification: Dense calcification can cause significant shadowing in OCT images, obscuring underlying structures. While IVUS can also be affected by calcification, its deeper penetration may allow for better visualization of the vessel beyond the calcified plaque in some cases [11].

### 6.2. Clinical Strengths

OCT: Excels in detailed plaque characterization [e.g., identifying TCFA, plaque erosion, thrombus], precise stent optimization [assessment of stent expansion, apposition, edge dissection, tissue prolapse], and guiding interventions in complex lesions where high-resolution detail is paramount [8].

IVUS: Is often preferred for assessing overall plaque burden, guiding stent sizing [by measuring EEL], evaluating vessel remodeling, and in cases of diffuse disease or heavily calcified lesions where deeper penetration is beneficial [11].

When used in combination, OCT and angiography provide detailed cross-sections that show the form and texture of plaques, the thickness of fibrous caps, and microscopic changes, making it easier to identify the lesion accurately [3]. This combined approach facilitates optimal stent placement and detection of problems during follow-up, potentially reducing the risk of stent blockage and other issues. The combination of OCT-guided PCI and angiographic information has been shown in clinical trials to lead to larger stent deployment and fewer cardiovascular problems than procedures guided by angiography alone [11]. Likewise, the fusion of angiographic and OCT data enables a clinician to better design their intervention, which may translate to more successful and safe operations [1].

Moreover, integration enables health specialists to evaluate the complex lesions more accurately, including bifurcations or calcified plaques that may not be well described with angiography only [3]. Distinct imaging methods allow for stratifying patients to identify the best treatment pathways, and they may even foster improved treatment outcomes of patients with coronary artery disease [11].

**Figure 4:**
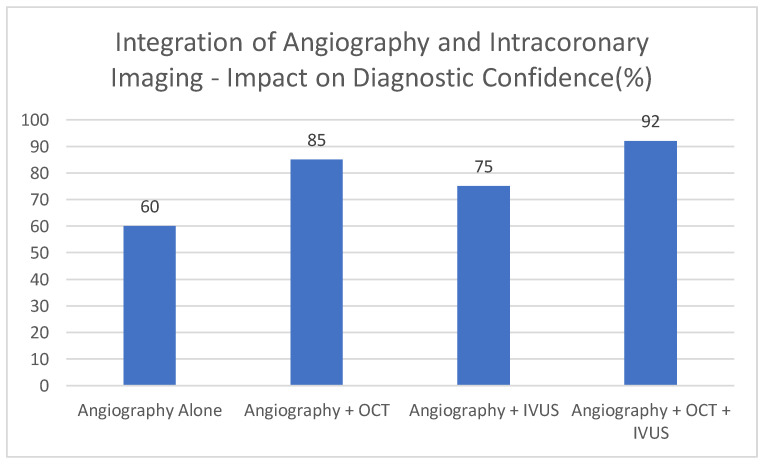
Integration of angiography and intracoronary imaging—impact on diagnostic confidence [%].

This graph [Figure 4] demonstrates exemplary data, which shows that OCT and IVUS, used in combination with conventional angiography, can help increase diagnostic confidence. This number is conceptual. Since angiography primarily provides luminal information, it is associated with the lowest illustrative percentage of cases diagnosed with high confidence [60%]. OCT, with its detailed visualization of the vessel wall and plaque features, is shown to increase diagnostic confidence to 85%. Combining IVUS into imaging procedures further increases illustrative confidence [75%], due to its deeper penetration and ability to provide information about vessel remodeling, despite its lower resolution compared to OCT. The descriptive confidence in providing the diagnosis rises to 92 per cent when angiography is accompanied by both the OCT and IVUS, indicative of the fact that a combination of the three can yield the most comprehensive overview of the coronary artery disease.

This integration technique increases physiology measurement precision, as it benefits the incorporation of anatomical data of functional measurement indicators, such as the instantaneous wave-free ratio [iFR] and fractional flow reserve [FFR] [22]. Fusion of intracoronary imaging and angiography data can help clinicians to better evaluate the amount and physiological significance of coronary atherosclerotic lesions.

## 7. Technological Advancements in OCT Imaging of Coronary Arteries

New OCT methods have remarkably extended the possibilities of diagnosing and treating coronary artery disease. The quality, speed, and depth penetration of pictures have been enhanced, with the clinicians being able to view the coronary architecture on an unprecedented scale [13]. The increasing potential of OCT goes beyond simple visualization to quantitative measurement of endothelial health, plaque characteristics, and stent placement, increasing diagnostic accuracy and informing treatment in coronary artery disease [24]. Moreover, the compatibility with IVI catheters allows OCT to be used in real-time during percutaneous coronary intervention, facilitating the placement of stents in the correct position, and the outcome of the procedure may become better.

The new technological developments have rung up OCT among the high ranks as a desirable means of coronary artery imaging due to the better image performance, high acquisition speed, and clinical flexibility. The technical advances made in catheter design have made it easier to maneuver through more tortuous and calcified blood vessels, and thus OCT has become an especially useful tool in complex cases [13]. The fast acquisition rate would enable clinicians to complete the exam in less time, with motion artifacts reduced and images being more readable [7]. The breakthrough is having the concept of OCT-based measuring techniques that measure fractional flow resistance [OCT-FFR] and the ability to derive both physiological information and images simultaneously, and in using only OCT technology [16]. These approaches use OCT-acquired lumen geometries and computational fluid dynamics insight to measure ischemia, focused on coronary flows and cross gradients prior to the surgery of each lesion [16].

Moreover, newer image processing algorithms have enabled characterizing plaque better and detecting such features as thin caps, macrophage infiltration, and the existence of microchannels within plaques [7]. These advances contribute to the application of OCT in monitoring PCI, evaluation of stent installation, and assessing patient responses to care. Artificial intelligence [AI] and machine learning are recent areas that can be applied to OCT image analysis to improve its accuracy and speed of diagnostics [13].

**Figure 5:**
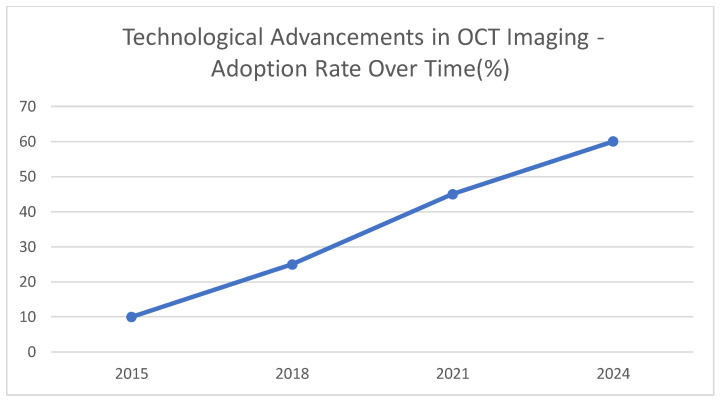
Technological advancements in OCT imaging—adoption rate over time [%].

This graph [Figure 5] represents illustrative data in the form of a rise in the utilization of OCT technology over a given time in PCI procedures. The projected and extended higher use between 2015 and 2024 reflects the greater appreciation of the advantages of such developments in clinical settings. The indicative adoption rate percentage is presented to increase tremendously between 2015 and 2024, by 60 percent. This growing application could indicate the advantages of more rapid swept-source OCT [SS-OCT] over cleaner images and enhanced ability to characterize plaque, as well as that of better catheter designs that allow access to more recalcitrant lesions. It is expected that OCT will be more actively deployed in everyday PCI procedures as it is constantly improving and proving to be more efficient in directing therapy and affecting long-term outcomes. With increased use of image-guided intervention, clinicians can gain more precision, which can provide an improvement in patient outcomes and safety.

Further improvements in the field of coronary imaging are expected to be achieved through the integration of the latest technological achievements in OCT imaging. The new innovations target extending the treatment utility offered by OCT in managing coronary artery disease. These innovations entail the improvement of image processing algorithms, artificial intelligence implementations, and the development of next-generation OCT devices [24]. To expand the scope of possible molecular and cellular correlates of coronary physiology and disease, additional advancements in coronary imaging applications of OCT would involve studies into new techniques of its use, including novel methods of molecular imaging and functional OCT imaging. Technological innovations allowing personalized and extremely targeted percutaneous interventions will also help in the diagnosis and treatment of coronary artery illnesses, thereby improving patient outcomes.

## 8. Comprehensive Utility of OCT for Coronary Artery Disease

OCT has been universally applied in many places in a clinical environment to optimize the management of coronary artery disease in clinical setups. The ability to view detailed features and characterize form and content with high resolutions helps to identify characteristics that could be consistent with vulnerable plaques that are likely to rupture, thereby facilitating the risk stratification [9]. Moreover, OCT is beneficial when assisting with positioning and placement of stents, which may enhance procedural success and eventually reduce incidences of procedure-related complications, such as stent thrombosis [8]. Additionally, intracoronary imaging areas, such as the application of OCT to in-stent restenosis patients, have already proved helpful in observational studies [25].

OCT may also be used in several functions of the management of CAD, namely, detection of disease, risk stratification, procedural guidance, and long-term follow-up. It has the potential to offer specific information on plaques, which may help it detect which ones may rupture easily and are missed during simple angiographic assessment [9]. Individualized risk assessment can be supported with these data, and clinicians can choose optimal medical and interventional strategies in patient treatment. Therefore, OCT has demonstrated decent value in PCI because of the precise placement of stent and their evaluation, which leads to potential decreased rates of stent thrombosis and restenosis [8]. OCT is also instrumental in assessing Myocardial Infarction with Non-Obstructive Coronary Arteries [MINOCA], as it may be used to identify candidate ruptured or eroded plaques or blood clots that angiography would have overlooked [5].

Second, OCT can be used to match anatomical outcomes and functional assessment, which would offer more context when making clinical decisions and predicting patient outcomes [16]. With this extended strategy of cardiovascular disease management, there is an opportunity to adjust any treatment scenario to a specific individual, which can provide a benefit and reduce the likelihood of adverse cardiac incidents [8]. The intrinsic implications of OCT technologies are only going to strengthen their core position in the diagnosis and management of heart diseases in the future [14].

**Figure 6:**
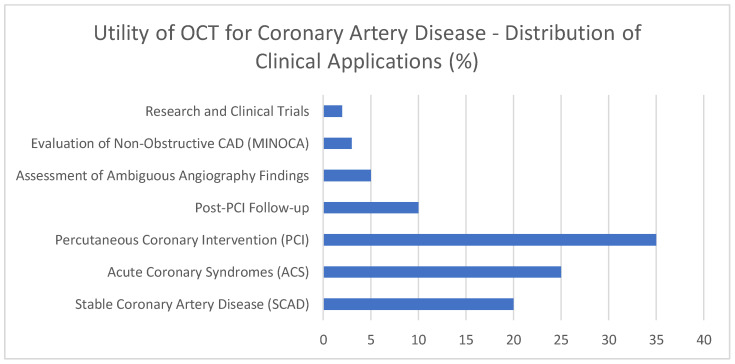
Utility of OCT for coronary artery disease—distribution of clinical applications [%].

This graph [Figure 6] contains exemplary data on the widespread suitability of Optical Coherence Tomography [OCT] in different scenarios involving coronary artery disease. This is just to give an illustrative figure. A significant portion of illustrative OCT procedures [35%] supports the accurate deployment of stents through PCI guidance, demonstrating the importance of this method. In addition, acute coronary syndromes [ACS] account for an illustrative 25% of the workload because OCT helps identify lesions that may require urgent treatment. In approximately 20% of cases, stable coronary artery disease (SCAD) incorporates OCT to identify and evaluate plaque types for proper risk assessment. For the 10% of patients who need post-PCI check-ups, OCT can help identify the causes of stent problems. Evaluating both uninterpretable angiography results [5%] and non-obstructive coronary artery disease [3%] shows that OCT offers benefits in challenging diagnostic situations. Only about 2% of the illustrative distribution is allocated to research and clinical trials, as these applications are quite specialized. This illustrative distribution of applications reveals that OCT is valuable for diagnosis, guiding procedures, and studying different aspects of coronary artery disease.

Pathological implications of OCT are also significant since they can be used to determine treatment options or the prognosis of the patient. The ability of OCT to provide detailed information about cardiac pathology enables it to be used in a different way each time, based on the individual features of a particular lesion. In general, OCT could be used to treat coronary artery disease in many ways, as mentioned, showing its usefulness in clinical practice by offering significant information that can be used to enhance patient outcomes and care.

## 9. Current Status of OCT-Based Physiological Assessment

OCT technology has evolved, with high penetration depth incorporated, great acquisition speed, and picture quality that enables accurate coronary functioning assessment [13]. The practicality of OCT has also recently been extended by determining the effectiveness of physiological parameters obtained by OCT, specifically a parameter called the optical flow ratio, in determining the degree of lesions, as well as in selecting treatment options in clinical practice “see Table 2 below” [1,4].

Measurement of FFR using OCT is a quickly developing, important approach to determine the blood flow of the heart. The OCT-FFR measures blood flow based on its excellent OCT image assessments and calculates the rate of blood supply to the lesions to indicate the state of the lesions [4]. This technique has already been well-paired to conventional wire-based FFR and has been associated with increased risks of target vessel failure [TVF] after PCI in patients with acute coronary syndromes [16]. OCT-FFR can be combined with the result of OCT images, the cap thickness, or the elements of plaques to improve the ability to predict further complications [4]. These encouraging results are encouraging, but the extensive clinical application of AI-based OCT-FFR in practice needs software standardization, proper staff education, and practical integration of AI-based OCT-FFR into existing clinical workflows. Interventional multi-center trials are in progress to understand the accuracy and how helpful OCT-FFR can be to a person who has a varied background in context [11].

Additionally, investigators also attempt to utilize other OCT-based physiological parameters in assessing the patient’s condition during PCI. The advances would allow closer examinations of lesions, possibilities to make better decisions about revascularization, and potentially better performance of patients with coronary artery disease [4,16].

This table provides a descriptive review of the current realities in the field of physiological assessment by using Optical Coherence Tomography [OCT]. Computationally generated OCT-FFR using Computational Fluid Dynamics on high-resolution 3D OCT with hyperemia simulation shows a moderate to good correlation with wire-based FFR. Its clinical use is, however, hindered by its complexity and the fact that it still needs substantial validation on a wide variety of patients and lesions. OCT-iFR is a current research field, studying flow across the diastolic phase as an easier mechanism of identifying functional significance that does not involve hyperemia. Adopting this method could simplify physiological assessments. In addition, preliminary studies analyze OCT Velocity Flow Reserve [OCT-VFR] and techniques to infer microvascular resistance [OCT-MRA] directly from OCT data. These approaches remain in the early stages and rely on further developments in image analysis and understanding how OCT features relate to coronary circulation. While OCT is primarily recognized for its detailed anatomical imaging, efforts to derive physiological insights are an active and promising field. The effective development and stringent testing of these OCT-based tools would create a great avenue through which the assessment of the structure and even functioning of coronary arteries would be improved, making the care of patients with coronary arteries much easier and simpler. However, before extensive clinical adoption, much research and technological advancement are needed.

OCT-based physiological markers are under development to become standardized to offer consistency and reproducibility across imaging technologies. The goals of standardization programs are to define guidelines on how to receive the image, processing methods, and interpretation parameters, to enhance the consistency of the OCT-based physiological tests [24]. The use of these standardized indicators appears to gain momentum as more interventional cardiologists and imaging specialists come to accept their use. As OCT-based physiological assessment continues to become more popular, it is expected that its integration into mainstream practice will further benefit patient care by enabling physicians with valuable information to formulate specialized care patterns and thus help to improve long-term outcomes in the management of coronary artery disease.

## 10. OCT-Based Fractional Flow Reserve

The OCT-based fractional flow reserve [OCT-FFR] combines high-resolution OCT anatomical features with computational fluid dynamics to present functional data that has served as a major milestone in the assessment of coronary physiology [5]. OCT-FFR adopts the OCT-generated luminal geometry, which is used to represent blood flow in the coronary arteries and present a functional and anatomically correct picture [18]. The method tries to provide a more detailed grading of the severity of the lesion with account taken of the hemodynamic significance, as well as the morphology of the lesion. OCT-FFR has the potential to improve the sensitivity of lesions in need of revascularization by optimizing lesion diagnosis, which can involve selective enrollment of patients to invasive procedures such as coronary artery bypass grafting [CABG], as well as percutaneous coronary intervention.

OCT-FFR measures coronary arterial ischemia on a per-lesion basis by measuring integrated OCT lumen measurements and computational fluid models. Unlike the usual wire-based FFR method in which pressure recording and vasodilator drug administration are required to elicit hyperemia, OCT-FFR is less invasive, faster, and tends to be less unpleasant to a patient [16]. This approach allows for the recording of anatomical and physiological information simultaneously in a single OCT scan, which could increase the effectiveness of diagnosis [5].

Clinical research has shown that OCT-FFR plays a part in predicting future events after PCI, particularly that low OCT-FFR is a risk factor that increases the likelihood of target vessel failure [TVF] and poor cardiovascular events [CVE] [4]. Furthermore, adding OCT-FFR to morphology, including TCFA, can enable future predictability of the patient having acute coronary syndrome [9]. By this determination of severity and vulnerability, the treatment can be tailored based on the patient, and by management of the risks, the patient can become better at managing the risks. The OCT-FFR has the potential to become a routine clinical variable to maximize PCI and manage coronary artery disease in patients, since algorithms and imaging have advanced. In the future, OCT-FFR is intended to be evaluated on more diverse groups of people and analyzed to determine whether it can be combined with artificial intelligence to become more practical and accurate [3,16].

Adding OCT-FFR values to the decision-making process of interventional cardiologists allows them to make decisions more effectively regarding revascularization because of the potential to prioritize treatment in the presence of the most severe coronary lesions. OCT-FFR offers a personalized way of care, by functional evaluation with anatomy, so that treatments are deployed on lesions causing potential hemodynamic compromise. The specificity of the personalized approach is to decrease the risk of poor outcome and increase efficiency of revascularization activities, proving that the revascularization must target in-sensors that are functionally significant [18]. Overall, OCT-based FFR can enhance coronary lesion management and revascularization approaches, and the management of coronary artery disease and patient outcomes.

## 11. Conclusions

Optical Coherence Tomography [OCT] has been of great benefit in gaining an insight into the coronary artery functioning and disease. Its ability to image at high resolution would enable an accurate evaluation of lesions, and this could be used to develop personalized treatment strategies by giving highly detailed information on the endothelium, plaque characteristics, and the optimization of stents. Anatomical detail is immensely powerful in OCT, but by virtue of its limitations, it requires a balanced approach, including, frequently, a complementary role towards other forms of imaging such as Intravascular Ultrasound [IVUS]. The combination of OCT and physiologic captures, especially by derived measures such as OCT-based fractional flow reserve [OCT-FFR] and optical flow ratio, is promising. The rationale behind these tools is to enhance the accuracy of diagnosis, which is performed by incorporating anatomical knowledge along with functional evaluation and, thus, further optimizing the treatment of coronary artery disease and even the patient outcomes. The advantages of OCT-guided PCI in enhancing the results of the procedures are demonstrated by randomized controlled trial evidence. With increased expansion and validation programs, there will be increasing and more advanced usage of OCT technology in efforts of percutaneous coronary intervention, which has a chance of delivering better patient care by offering conclusive, OCT-tinged analyses. Further developments in complex image processing, incorporation of artificial intelligence, and new functional applications will further increase the use of OCT in the emerging milieu of cardiovascular diagnostics and therapeutics.

## Figures and Tables

**Table 1:** Physiological assessment of coronary lesions.

Parameter	Technique	Clinical Utility
FFR	Invasive pressure wire (hyperemia)	Hemodynamic significance of stenosis; guides revascularization in stable CAD.
IFR	Invasive pressure wire (diastole)	Alternative to FFR; assesses lesion significance without hyperemia.
CFR	Doppler flow wire (hyperemia)	Microvascular function + epicardial stenosis; identifies microvascular disease.
IMR	Doppler flow wire (adenosine)	Quantifies microvascular dysfunction; prognostic value.

**Table 2:** OCT-based physiological assessment.

OCT-Derived Parameter	Methodology	Invasive Correlation	Clinical Status
OCT-FFR	CFD on 3D OCT (simulated hyper.)	Moderate to Good	Research: Limited clinical use due to complexity and validation needs.
OCT-iFR	CFD on 3D OCT (diastolic phase)	Emerging	Early research: Potential advantage of hyperemia-free assessment.
OCT-VFR	Velocity/Lumen analysis	Preliminary	Very early stage: Investigating flow dynamics from OCT images.
OCT-MRA	Microvascular assessment via OCT	Exploratory	Conceptual: Investigating OCT’s ability to infer microvascular function.

Note: This table represents a factual overview depending on the understanding of the subject now.

## Data Availability

Not applicable.

## Abbreviations

OCT—Optical Coherence Tomography, CAD—Coronary Artery Disease, FFR—Fractional Flow Reserve, iFR—Instantaneous Wave-Free Ratio, CABG—Coronary Artery Bypass Grafting, OCT-FFR—OCT-based Fractional Flow Reserve, PCI—Percutaneous Coronary Intervention, CT—Computed Tomography.
